# Association between Fish Consumption and Prefrontal Function during a Cognitive Task in Male Japanese Workers: A Multi-Channel Near-Infrared Spectroscopy Study

**DOI:** 10.1371/journal.pone.0123972

**Published:** 2015-04-28

**Authors:** Shenghong Pu, Kazuyuki Nakagome, Takeshi Yamada, Hiroshi Matsumura, Katsutoshi Yokoyama, Koichi Kaneko, Yoichi Kurosawa

**Affiliations:** 1 Division of Neuropsychiatry, Department of Brain and Neuroscience, Tottori University Faculty of Medicine, Yonago, Tottori, Japan; 2 Department of Psychiatry, National Center of Neurology and Psychiatry Hospital, Tokyo, Japan; 3 Division of Health Administration and Promotion, Department of Social Medicine, Faculty of Medicine, Tottori University, Yonago, Tottori, Japan; Chiba University Center for Forensic Mental Health, JAPAN

## Abstract

The purpose of this study was to examine the relationship between fish consumption and prefrontal function during a cognitive task in male Japanese workers. The study included 208 male workers who underwent medical health examinations 3 months after a change in their work assignment. We measured the hemoglobin concentration changes in the prefrontal region during working memory tasks using 52-channel near-infrared spectroscopy. The frequency of fish consumption was calculated on the basis of the subjects’ self-reported customary intake frequency over the previous 3 months. A significant positive relationship was observed between fish consumption and left dorsolateral prefrontal function during a working memory task. To our knowledge, this is the first study to report an association between fish consumption and functional cortical activity with an ample sample size, suggesting that fish consumption modulates functional activity in the left dorsolateral prefrontal cortex.

## Introduction

Over the years, fish consumption has been linked to multiple health benefits [1–5]. Fish is a major dietary source of long-chain omega-3 polyunsaturated fatty acids (PUFAs), especially eicosapentaenoic acid (EPA) and docosahexaenoic acid (DHA), which are critical for brain structure and functioning [6]. EPA and DHA have also been shown to benefit mental health [7,8]. Dietary consumption of EPA and DHA is important because the body cannot synthesize these substances de novo, although limited amounts can be synthesized from α-linolenic acid [9].

Low intake of omega-3 PUFAs may lead to neurocognitive deficits, anxiety, aggression, and depression [10,11]. Considerable evidence suggests that insufficient intake of omega-3 PUFAs and low concentrations of EPA and DHA are associated with depressive disorders [12–22]. Numerous epidemiologic and dietary studies confirm that supplementation of omega-3 PUFAs is inversely related to the prevalence and severity of depression [21,23,24]. Furthermore, several cross-national analyses reported highly significant inverse relationships between fish consumption and the prevalence of major depressive disorder [13,16,25], postpartum depression [17], and bipolar depression [26]. Deficiency of omega-3 PUFAs has also been linked to several other major neurobehavioral disorders, including attention deficit hyperactivity disorder [27] and schizophrenia [28].

Recently, it has been acknowleged that depression is associated with cognitive deficits presumably based on functional impairment in the prefrontal cortex (PFC) [29]. Therefore, we speculated that the low fish consumption as well as deficiency of omega-3 PUFA may be one of the vulnerable factors for inducing depression by its adverse effect on prefrontal function. To date, few studies have reported an association between omega-3 PUFA intake and brain functional activity [30,31], and to our knowledge, no studies have evaluated fish consumption and brain functional activity with an ample sample size. Although the health benefits of PUFAs have been extensively studied for years, most research has focused on the effects of PUFA supplement intake rather than that of direct fish consumption. In addition, most studies have been conducted in infants and the elderly rather than in young and middle-aged populations.

In Japan, fish is an important staple of the traditional diet. However, fish consumption in Japan has been gradually decreasing in recent years, particularly among young people, in keeping with the westernization of their diets [32]. The prevalence of major depression in Japan has historically been relatively low as compared to that in Western countries [33,34]. However, the prevalence of mood disorders, including depression, has increased over the last decade, especially among the young and middle-aged population [35–38]. Although this increase is considered a consequence of the recession in Japan [39], taking into account the previous studies [13,16,25], which showed a high consumption of fish could be correlated with a lower prevalence of major depression, dietary changes, mainly among young people, may also be a contributing factor.

The present study investigated the effects of fish consumption on brain functional activity in young-to-middle-aged male Japanese workers. Brain functional activity was assessed via a working memory task previously shown to activate the PFC, while local cerebral blood volume was measured in the PFC using multi-channel near-infrared spectroscopy (NIRS), a non-invasive brain imaging technique [40–42]. We hypothesized that higher fish consumption is associated with higher prefrontal function level.

## Methods

### Subjects

The subjects were workers from a single branch of a company (listed in the first section of the Tokyo Stock Exchange) who underwent medical health examinations at Tottori University Hospital 3 months after a change in work assignment. Between April 2008 and March 2013, 313 subjects aged between 21 and 55 years underwent the examination.

In this study, the exclusion criteria were the following: (1) current use of psychotropic drugs such as antidepressants, mood stabilizers, and antipsychotics; (2) history of head injury; (3) current or past history of psychiatric illnesses including substance and/or alcohol abuse; and (4) left-handedness. Only 10 out of 313 subjects were female; therefore, only the male subjects were included in the study. This study was approved by the Ethics Committee of the Tottori University. All the participants gave written informed consent in accordance with the Declaration of Helsinki after a complete explanation of the study. All the participants were right-handed with criteria of more than 80% by the Edinburgh Inventory Index [43].

### Self-report measures

Subjects completed the Beck Depression Inventory (BDI), which is a self-reported measure designed to assess current depressive symptomatology [44], and Social Adaptation Self-evaluation Scale (SASS) [45,46] was used to evaluate social functioning. Participants completed the questionnaires within 1 week before the medical examination.

Fish consumption was scored on the basis of the subjects’ self-reported customary intake frequency during the previous 3 months using the following scale: (0) almost never, (1) 1–2 times/month, (2) 1–2 times/week, (3) 3–4 times/week, and (4) almost every day [47]. Since the socioeconomic status (SES), assessed on the basis of the income, occupation, and education level of the individual, is known to influence fish consumption [48,49], information on education level (number of years) was also collected to adjust for the influences of SES. Other factors influencing SES were considered negligible because the subjects worked in the same company under an identical wage structure.

### Measurement of plasma EPA and DHA concentrations

Blood samples were obtained between 9:00 am and 11:00 am on the day of the medical mental health examination. Plasma concentrations of EPA and DHA (μg/ml) were measured at SRL Co., Ltd. (Tokyo, Japan). Briefly, total lipids were extracted with chloroform:methanol, 2:1 (vol/vol). Methyl esterification of fatty acids was performed using potassium methoxide (0.4 mol/l) and boron trifluoride-methanol (14 wt/vol%). The composition of fatty acids was analyzed using gas chromatography (Gas Chromatograph GC-17A equipped with Auto Injector AOC-17 and Data Processor C-R7A; Shimadzu Co, Kyoto, Japan), and the percentages of phospholipid fatty acids were determined for EPA (20:5n-3) and DHA (22:6n-3) [50].

### Fronto-temporal function

Fronto-temporal function was assessed on the basis of measurement of hemoglobin concentration changes in the fronto-temporal regions during a cognitive task using 52-channel NIRS.

### Cognitive task

**Fig 1:**
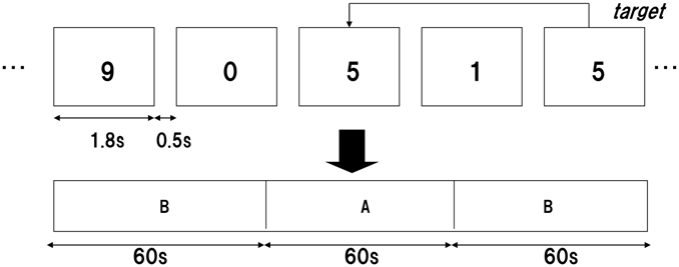
The task design of the 2-back task. A: Activation condition: 2-back. B: Baseline condition: 0-back, “9” as the target.

**Fig 2:**
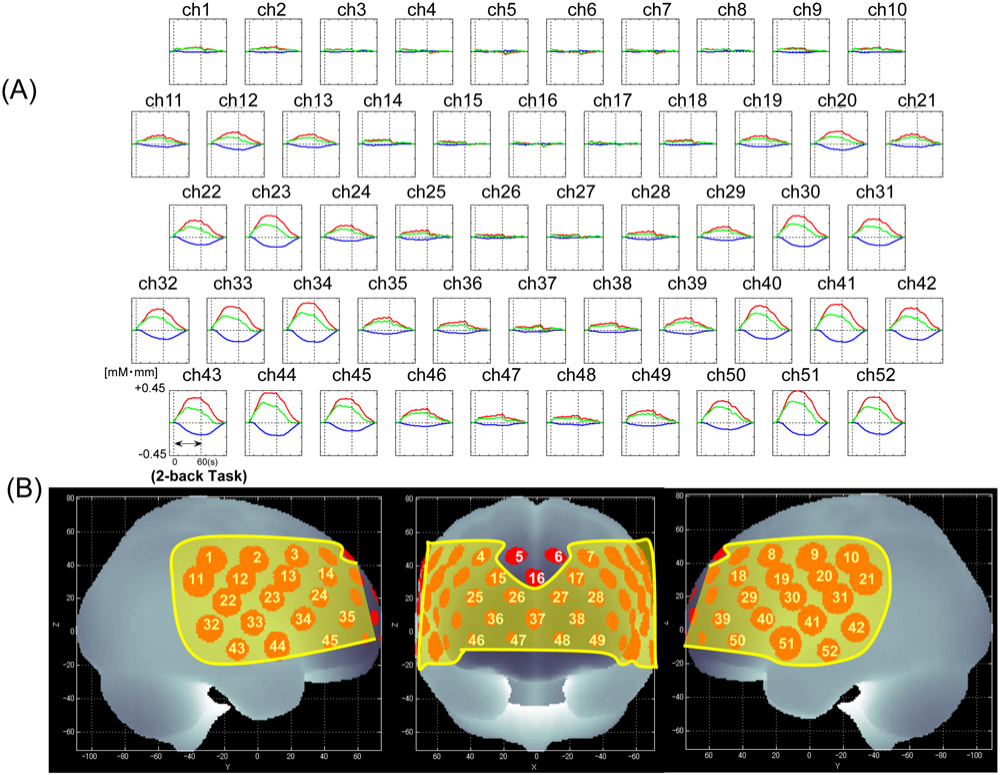
NIRS signal change between 2-back task and 0-back task (baseline). (A) Grand average waveforms in male workers (N = 208). Oxy-, deoxy-, and total-hemoglobin concentration changes during cognitive activation are presented as grand average waveforms in 52 channels in red, blue, and green lines, respectively. (B) Brain area in yellow corresponds to the NIRS channels, which showed significant increases during the 2-back condition compared with the pre-task 0-back condition in the mean [oxy-Hb] changes in the subjects (52 channels-FDR-corrected p < 0.05). The locations of NIRS channels were probabilistically estimated and anatomically labeled in the standard brain space in accordance with Tsuzuki et al. [58].

**Fig 3:**
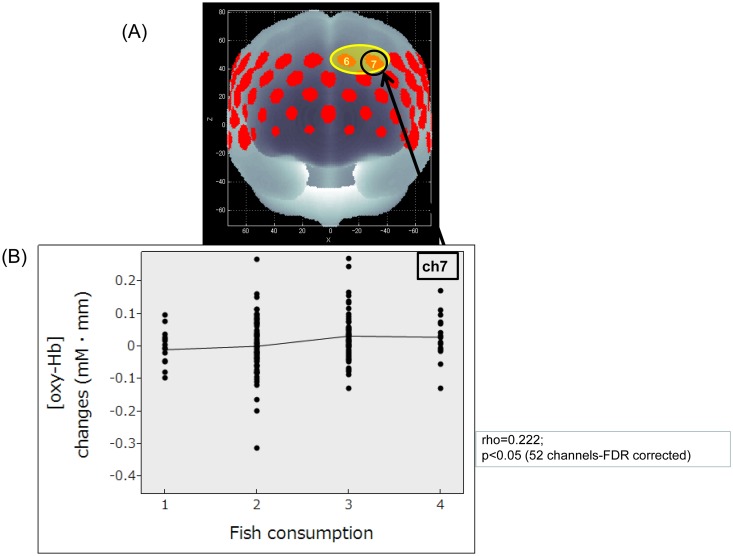
The figure shows the relationship between [oxy-Hb] changes and fish consumption levels. Each number shown for fish consumption levels indicates the subjects’ customary fish intake frequency during the previous 3 months. No subject reported “almost never.” The frequency “1–2 times/month” was coded as 1; “1–2 times/week,” as 2; “3–4 times/week,” as 3; and “almost every day,” as 4. (A) Brain area in yellow corresponds to the NIRS channels; the [oxy-Hb] changes showed significant correlation (Spearman rank correlation; 52 channels-FDR-corrected p < 0.05) with fish consumption levels. (B) Scatter diagram at ch7 (left DLPFC; Spearman rank correlation coefficient; rho = 0.222; p < 0.05, FDR-corrected). The locations of NIRS channels were probabilistically estimated and anatomically labeled in the standard brain space in accordance with the study of Tsuzuki et al. [58].

We used a 2-back task with a blocked periodic baseline-activation-baseline (Fig 1) design to activate brain regions specialized for maintenance components of verbal working memory, as originally described by Cohen et al. [51]. Two contrasting conditions were visually presented in 60-s periods to subjects on a computer screen placed approximately 1 m away from the subjects’ eyes. During the period of the baseline (B) condition, subjects viewed a series of figures (0–9), which appeared one at a time. Subjects were asked to press a button with their right index finger whenever the figure “9” appeared (0-back). During the period of the activation (A) condition (2-back), subjects again viewed a series of figures (0–9) and were asked to press a button with their right index finger if the currently presented figure was the same as that presented 2 trials previously (e.g., 5-1-5 but not 2-6-3-2 or 2-7-7). The working memory task consisted of a 60-s pre-task period (baseline [B] condition), a 60-s 2-back task period (activation [A] condition), and a 60-s post-task period (baseline [B] condition). Each period comprised 25 stimuli (5 targets, stimulus duration = 1.8 s, stimulus onset asynchrony [SOA] = 2.3 s). Behavioral performance for the 2-back task was monitored and measured in terms of reaction time (RT) to target figures and sensitivity A′ [52]. Sensitivity A′ is an index of information processing ability using both “hit rate (HR)” and “false alarm rate (FAR)” for calculation, which is expressed as below:

A'=0.5+(HR−FAR)(1+HR−FAR)/4HR(1−FAR)

High A′ implies high information-processing ability. All subjects received a brief period of identical training to ensure that they understood the rule of the task prior to measurement.

### NIRS measurements

The 52-channel NIRS machine (ETG-4000, Hitachi Medical Co.) measures relative changes of [oxy- Hb] and [deoxy-Hb] using two wavelengths (695 and 830 nm) of infrared light on the basis of the modified Beer—Lambert law [53]. In this system, these [Hb] values include differential path length factor (DPF). Zhao et al. [54], using a Monte Carlo simulation, reported that the estimated DPF variation in the forehead region of adult humans was considered roughly homogeneous. The distance between pairs of source-detector probes was set at 3.0 cm, and each area located at the mean distance between the source-detector pairs was defined as “channel.” The machine measures points at a depth of 2–3 cm from the scalp; that is, the surface of the cerebral cortex [55,56]. The probes of the NIRS machine were fixed with thermoplastic 3 × 11 shells, with the lowest probes positioned along the T3-Fp1-Fpz-Fp2-T4 line according to the International 10–20 System used in electroencephalography. The arrangement of the probes enabled the measurement of [Hb] values from bilateral prefrontal and superior temporal cortical surface regions. The correspondence of the NIRS channels and the measurement points to the cerebral cortex were confirmed by a multi-subject study of anatomical craniocerebral correlation [57] and was presented on the basis of the results of the virtual registration method [58].

The rate of data sampling was 0.1 s. Data were analyzed using the “integral mode”; the pre-task baseline was determined as the mean over a 10-s period immediately prior to the task period, and the post-task baseline was determined as the mean over the last 5 s of the post-task period; linear fitting was applied to the data between these 2 baselines with the aim of reducing the effect of presumably task-unrelated low frequency activity. A moving average method using a window width of 5 s was applied to remove any short-term motion artifacts. Because we could not remove all artifacts in this way, we applied automatic rejection of data with artifacts separately for each channel [41,42,59].

### Statistical analysis

Statistical analyses were performed using SPSS Statistics 19.0 software (Tokyo, Japan).

### Hemodynamic response data

For the analysis of the hemodynamic response data, [Hb] concentrations of [oxy-Hb], [deoxy-Hb], and [total Hb] for each channel were averaged for the 2 time segments (pre- and post-task baseline and task period). We focused on [oxy-Hb] concentrations, since [oxy-Hb] change (task period − pre- and post-task baseline period) is assumed to more directly reflect cognitive activation than [deoxy-Hb] change as shown by a stronger correlation with blood-oxygenation level—dependent signal measured by fMRI [60]. We thus first compared the mean [oxy-Hb] levels between the task period and the baseline using Student’s paired *t*-test in each channel.

### Relationship between cortical function and fish consumption

First, to explore the relationship between fish consumption and fronto-temporal cortical activation, we compared the mean [oxy-Hb] changes between the task period and the baseline between high and low fish consumption levels. Next, Spearman rank correlation coefficients were calculated for testing the correlations between the mean [oxy-Hb] changes between the task period and the baseline and fish consumption levels for each NIRS channel, where a significant difference in the mean [oxy-Hb] changes was observed between different levels of fish consumption. We set the value of q specifying the maximum false discovery rate (FDR) to 0.05, so that there were no more than 5% false-positives on average [61]. In addition, multivariate analysis was conducted using generalized linear model to examine the relationship between mean [oxy-Hb] changes and fish consumption levels using mean [oxy-Hb] changes as a dependent variable and fish consumption levels as well as other possible confounding variables, such as age, education levels, SASS total scores, BDI total scores, sensitivity A’, plasma EPA and DHA concentration, as independent variables.

## Results

Consenting participants who were excluded were as follows: 2 out of 303 subjects were taking psychotropic drugs, 23 had a history of head injuries, and 32 were left-handed. No participant had current or past history of substance/alcohol abuse. Twenty-four of 246 were diagnosed as having major depressive episode and 14 subjects had a past history of depression (n = 38, depression subgroup) according to DSM-IV criteria. The data obtained from 208 male healthy subjects were submitted to analysis accordingly.

First we found significant increase in the mean [oxy-Hb] levels during the task period compared with the baseline in all channels but ch5, 6 and 16 (FDR-corrected p: 0.001–0.047) (Fig 2).

As the number of persons in self-reported fish consumption level 1 (n = 17) and 4 (n = 15) were much smaller than those in level 2 (n = 99) and 3 (n = 77), to maintain the statistical power, we decided to compare the increase in the mean [oxy-Hb] levels compared with the baseline between the two subgroups by combining level 1 and 2 (low consumption subgroup), and level 3 and 4 (high consumption subgroup) (Table 1). There were significant differences in the [oxy-Hb] changes between the 2 subgroups in the channels located in the left DLPFC region at 2 channels (ch6, ch7: p < 0.05; FDR-corrected). Also, the mean [oxy-Hb] changes positively correlated with fish consumption levels in the 2 channels (ch6: rho = 0.23, p < 0.05; ch7: rho = 0.22, p < 0.05; FDR-corrected), located in the left DLPFC) region (Fig 3).

**Table 1:** Comparison between high-fish consumption subgroup and low-fish consumption subgroup.

	High-fish consumption (n = 92)	Low-fish consumption (n = 116)	Group difference
			*P*-value
Fish consumption	3.15	1.85	<0.0001
EPA	81.6±55.73	63.2±65.60	<0.05
DHA	136.5±52.58	107.0±40.37	<0.001
Age (years)	38.8±12.11	32.3±11.08	<0.001
Education (years)	12.7±1.55	12.9±1.61	0.31
BDI	3.1±3.86	3.9±4.86	0.15
SASS total	39.3±8.05	36.9±7.12	<0.05
interpersonal relationships	16.6±3.67	15.7±3.58	0.086
interest and motivation	15.0±4.09	13.6±3.74	<0.05
self-perception	7.7±1.60	7.6±1.37	0.44
Task performance: reaction time (RT; ms)	609.1±157.20	614.9±149.98	0.79
(2-back task) sensitivity A'	0.988±0.033	0.987±0.033	0.95

Multivariate analysis applying generalized linear model was conducted using [oxy-Hb] changes (ch6, 7) as a dependent variable and fish consumption levels, age, education levels, SASS total scores, BDI total scores, sensitivity A’, plasma EPA and DHA concentrations, as independent variables. As a result, the relationship between mean [oxy-Hb] changes (ch6, 7) and fish consumption levels remained significant (ch6: beta = 0.015, Wald chi-square = 5.20, p = 0.023; ch7: beta = 0.016, Wald chi-square = 4.36, p = 0.037).

## Discussion

A significant positive relationship between fish consumption levels and the left DLPFC function during a working memory task was observed in this study.

Although previous studies indicated that dietary consumption of EPA and DHA through fish may lead to high brain functional activity [30,31], in the present study, we did not find a significant correlation between the mean [oxy-Hb] changes in the left DLPFC and plasma EPA or DHA concentrations. Moreover, fish consumption levels were significantly related to the mean [oxy-Hb] changes in the left DLPFC independent of plasma EPA or DHA concentrations along with other possible confounding factors in the multiple regression analysis, suggesting it is unlikely that omega-3 PUFAs are the mediators between fish consumption levels and PFC function.

In a study by McNamara et al. [30] utilizing fMRI, DHA supplementation for 8 weeks resulted in increased activation of the dorsolateral PFC during a sustained attention task in male schoolchildren between the ages of 8 and 10 years compared with the placebo group. A study by Jackson et al. [31] utilizing NIRS, found that, compared with placebo, DHA-rich FO (DHA 5:1 EPA) supplementation for 12 weeks resulted in increased activation of the PFC during cognitive task in an adult population.

Actually, McNamara et al. [30] found a significant correlation between erythrocyte DHA composition and DLPFC activation. The discrepancy between the findings in their study and ours may be caused by the difference in the study design, subjects’ age and methodology of measuring the omega-3 PUFAs levels. The study design in McNamara et al. [30] was a relatively short-term intervention lasting for 8 weeks, which was in contrast to our epidemiological study design, and was conducted with healthy boys aged 8–10 years. Also, in the present study, we measured plasma EPA and DHA concentrations instead of erythrocyte compositions. Moreover, in a recent study by Raji et al. [62], dietary consumption of fish was associated with larger grey matter volumes independent of omega-3 PUFA estimates, which is in agreement with the present finding. Although the causal relationship between fish consumption and PFC function is unclear, a general healthier life style associated with fish dietary and with relatively high interest and motivation, as well as other components contained in fish or in the diet of individuals who frequently consume fish may lead to improvement in brain function.

As can be seen in Fig 2, the activation observed in the channels that showed significant correlation with fish consumption levels was, in average, very low. The finding may imply that the left DLPFC was in fact deactivated in those with relatively low fish consumption in comparison with those with high fish consumption. This may have occurred due to the exceedingly high load on cognitive function in those with low fish consumption leading to decreased interest and motivation level to 2-back task. We may consider that those with high fish consumption show stronger activation while they are engaged in a highly demanding task compared to the activation elicited by a simple task, whereas this was not true for those with low fish consumption.

The activated region in the present study extended far beyond DLPFC, which was much broader than that observed in fMRI studies using the same n-back task. However, in healthy subjects, Owen et al. [63] also suggested that the n-back task activated a wide bilateral network consisting of VLPFC and DLPFC, frontal poles, lateral premotor cortex, dorsal cingulate and medial premotor cortices, and medial and lateral posterior parietal cortices. In the present study, significant activation was indicated even in the superior temporal cortex. The seemingly extensive region of activation may have been caused by the poor spatial resolution, which is one of the shortcomings of NIRS measurement. Another possibility is the intermingling effect of extracranial hemodynamic changes such as skin blood flow in the measurement data, which has raised a question as to what extent NIRS signals reflect hemodynamic changes in the brain [64]. Although the impact of the extracranial artifacts, including their significance and generality, has not been clarified, Sato et al. [65] demonstrated that temporal changes in the NIRS signals in the activated area were significantly correlated with the BOLD signals in the gray matter rather than the extracranial BOLD signals or skin blood flow measured with a laser Doppler flowmeter.

There are several limitations to our study. The study design was cross-sectional, and furthermore, the present findings were obtained from only male workers because most of the subjects recruited from the mental examination were male, and also the kind of occupation that the workers were engaged in differed between sexes. In addition, EPA and DHA concentrations were measured in the plasma rather than the erythrocyte membrane, which has been shown to provide a more reliable estimate of the fatty acid composition in the brain region [66]. Another potential limitation is related to the functional integrity and resilience of cortical neurons, which are mediated in part by cortical DHA composition [30]. This might have caused a rather weak association between omega-3 PUFAs concentrations and PFC function. Finally, although a significant correlation was obtained between fish consumption levels and the mean [oxy-Hb] changes in 2 channels after 52-channels FDR correction, the rho values (0.22–0.23; n = 208) were generally low. The finding suggests that there may be a number of other confounding factors to be taken into consideration. For instance, not only the intake frequency but also kinds of fish and/or intake amounts should have been taken into consideration along with other diets. In future studies, we need to perform multivariate analyses including other variables not considered in the present study that may influence fish consumption levels and/or hemodynamic response in the PFC. Thus, our findings may have inferential and generalizability limits. Additional studies with a larger sample size in the general population are needed to further elucidate the relationship between fish consumption levels and prefrontal function and its mediators.

## Conclusions

This study provides evidence of a significant positive relationship between fish consumption and the left DLPFC function during a working memory task. Furthermore, [oxy-Hb] change during working memory task was significantly larger among subjects with high fish consumption than in those with low consumption, a finding that was not explained by differences in task performance. To our knowledge, this is the first study to report an association between fish consumption and functional cortical activity with an ample sample size, suggesting that fish consumption modulates functional activity in the left DLPFC.
